# Effect of Mini-Trampoline Physical Activity on Executive Functions in Preschool Children

**DOI:** 10.1155/2018/2712803

**Published:** 2018-05-10

**Authors:** Xu Wen, Ying Zhang, Zan Gao, Wei Zhao, Jiang Jie, Li Bao

**Affiliations:** ^1^Department of Physical Education, College of Education, Zhejiang University, Hangzhou, China; ^2^Hangzhou College of Early Childhood Teacher Education, Zhejiang Normal University, Hangzhou, China; ^3^School of Kinesiology, University of Minnesota-Twin Cities, Minneapolis, MN, USA; ^4^The Second Kindergarten of Cuiyuan, Xihu District, Hangzhou, China

## Abstract

The study investigated the effect of mini-trampoline physical activity on the development of executive functions (EF) in Chinese preschool children. Fifty-seven children aged 3–5 were randomly assigned to an intervention group (*n* = 29) and a control group (*n* = 28). The children in the intervention and control group had the same classes and care service in the preschool, but children in the intervention group had an extra 20 min of trampoline training after school for 5 school days per week in the 10-week intervention. Spatial conflict arrow (SCA), animal Go/NoGo (GNG), working memory span (WMS), and flexible item selection (FIS) were used to assess children's EF before and after the intervention. Results revealed that no significant differences emerged in the SCA, GNG, WMS, and FIS tests between two groups postintervention. Findings indicated that a 10-week trampoline PA training may not be sufficient to trigger the improvement of preschool children's EF. Future research with larger representative samples is warranted to discern the dose-response evidence in enhancing young children's EF through physical activity.

## 1. Introduction

It was widely accepted that physical activity (PA) plays a key role in the growth and development of children. The benefits of PA for preschool children may include but are not limited to controlling weight status and blood pressure, developing motor skills, and improving psychological wellbeing [1–4]. Over the past two decades, inspired by the findings in neuroscience and embodied cognitive science, many studies have been conducted to investigate the effects of PA programs on the cognitive functions in children [5–7]. Most of these studies have favored the positive correlations among PA and cognitive functions.

Executive function (EF) refers to the advanced cognitive ability of coordinating and controlling a set of cognitive processes for the attainment of a specific goal [8]. The EF of preschool children, which may affect the subsequent development of their academic achievement [9, 10] and social interaction [11, 12], is one of the important components in individual development. Recent studies indicated that PA intervention may exert beneficial effects on children's EF. Experimental research suggested that both acute and chronic aerobic exercises could effectively improve children's EF [13]. However, the findings have yet to be generalized to school or other naturalistic environments as some studies were conducted in laboratory setting [5]. Furthermore, studies also suggested that not all forms of physical activity equally promote EF [13]. It was reported that children in a 4-week foreign language vocabulary program with integrated PA achieved better learning outcomes than children in conventional condition [14]. However, a randomized controlled trial (RCT) indicated that the attention and working memory of children were not significantly improved following a one school year intervention [15]. Therefore, additional studies are needed to strengthen the evidence base for intervention programs of PA toward the development of EF and related outcomes.

Trampoline, a type of gymnastics and also known as “air ballet,” is an athletic sport very popular in Chinese preschool children, which uses acrobatic skills to rebound from trampoline. During mini-trampoline PA, children need to continuously respond to a constant change in gravity and adjust their body posture for the next rebound [16]. Trampoline exerts remarkable effects on the sense organs and nervous system of children (e.g., position sense, visual sense, proprioceptive sense, and motor control) and their physical fitness (e.g., strength, balance, and coordination). Previous research has indicated that a 12-week trampoline training intervention program of individualized daily 20-minute sessions can significantly improve the motor and balance ability of school-aged children [17]. Therefore, trampoline as a popular physical activity among preschool children may be considered an ideal exercise and is beneficial in improving the EF of preschool children. The current study aimed at investigating the effects of mini-trampoline PA program on the development of EF in preschool children.

## 2. Methods

### 2.1. Study Design and Participants

Preschool children aged 3-4 years were recruited from a preschool in the City of Hangzhou, China, in the current study. The teachers in the preschool and the parents of the children were invited in an introduction meeting, during which the research objectives and procedures were briefed. The parents agreed to have their children participate in the study, signed a written consent form, and completed a questionnaire reporting children's mental and physical health status. The study was approved by the ethics committee of Hangzhou College of Early Childhood Teacher Education, Zhejiang Normal University. Inclusion standards for children were as follows: (1) without psychological and mental illness or disorder; (2) without physical disability; and (3) normal vision.

### 2.2. Sample Size Calculation

According to the data of our pilot study, the mean accuracy and standard deviation of EF tests including Spatial Conflict Arrows test, Working Memory Span test, and Flexible Item Selection test were 75% and 15%, respectively. The accuracy was expected to increase by 12%. For this effect size with a power of 0.80 at alpha 0.05, a sample size of 25 is needed [18]. If dropout rate was estimated to be 10%, 28 participants are needed for each group.

### 2.3. Randomization and Blinding

**Figure 1:**
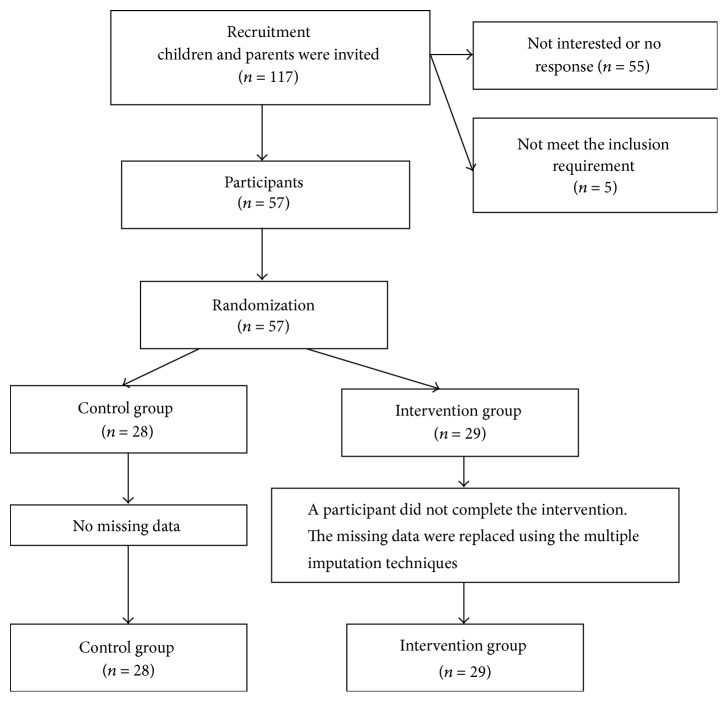
Flowchart of children from enrolment and allocation.

As shown in Figure 1, 117 preschool children were assessed for eligibility, but 55 parents of the children declined to participate and 5 children did not meet the inclusion criteria. A total of 57 children (31 boys and 26 girls) with a mean age of 4.40 years (SD = 0.29) participated in the study. The children were randomly assigned to either an intervention group (29 children) or a control group (28 children) with the use of a random number generator. One boy had not completed the intervention program (see Figure 1). Randomization and allocation to the experiment and control groups were conducted after pretest of EF by a technician. The researchers were not involved in the allocation to treatment group or control group. The measurement of EF was conducted by a trained researcher who was blinded to the group assignment. The primary researchers were not involved in the measurement work.

### 2.4. Procedures

The current study included the following three phases: (1) EF pretest, (2) a 10-week intervention, and (3) EF posttest. First, all children were invited to participate in the EF pretest. Second, during the following 10 weeks, the children in the intervention and control group shared the same classes and care service in the preschool, but children in the intervention group had an extra 20 min of trampoline training program after school (starting from 15:00 to 15:20) for 5 school days per week. The mini-trampoline PA intervention was conducted by a physical education teacher. The children received a 20-minute trampoline PA at his or her own minitrampoline surrounded with safety nets. The safety rules recommended by the American Academy of Pediatrics were followed to prevent sport injuries [19]. Finally, the EF posttest was conducted with the same instruments after the 10 weeks of intervention.

### 2.5. Measurement

All tests were completed by a trained tester. The EFs of each child were obtained by the tester individually with EF test software installed in a tablet computer. Each test was conducted in a private, quiet, and bright classroom. Prior to the formal test, the tester detailed the rules of the test to each child, and then each child took the formal test when he or she was fully trained and familiar with the test rules.

#### 2.5.1. Inhibitory Control

Spatial conflict arrow (SCA) task was modified by Willoughby and his colleagues in 2012 [20] based on the spatial conflict test introduced by Gerardi-Caulton in 2000 [21]. The SCA task was widely applied to assess the EF of 3–6 years old children [20, 22] and was used to measure the inhibition control of children. The details of the test were introduced in a previous study [20]. During the test, the children were asked to use their left hand to click the green dots on the left when the arrow pointed to the left and right hand to click the green dots on the right when the arrow pointed to the right. For items 1–8, the arrows were displayed in the center, which could help children be familiarized with the rules. For items 9–22, the arrows were displayed laterally, with the arrows pointing toward the left on the left side of the screen (above the left green dots) and toward the right on the right side of the screen (above the right green dots). For items 23–36, the arrows were displayed contralaterally, with the arrows pointing to the left on the right side of the screen and pointing to the right on the left side of the screen. Items presented contralaterally required inhibitory control from the previously established response.

Animal Go/NoGo(GNG) task, a test to measure the inhibitory control of preschool children, was developed in 2012 [20] based on the classic Go/NoGo task [23]. In the GNG test, children needed to click the green button at the fastest speed when they see animals on the screen except when a pig was displayed. The reaction time and accuracy in the SCA and GNG tests were recorded to reflect the inhibitory control of children.

#### 2.5.2. Working Memory

Following the principles and methods applied in the measurement of working memory in 1980s [24, 25], working memory span (WMS) task was developed in 2012 [20] and was adopted to measure children's working memory in this study. In detail, children saw some animals and a colored dot in a house (or several houses). The children needed to name the animals and color (all animals and colors were taught to the children prior to the test). Notably, only the outline of the house/houses would appear on the screen. The tester would require the children to recall which animal lived in the house/houses. The details of the task were described in a previous study [20]. The accuracy of WMS test was recorded.

#### 2.5.3. Cognitive Flexibility

Flexible item selection (FIS) task was used to measure cognitive flexibility of children. The test, which was designed by Jacques and Zelazo in 2001 [26], has been frequently used in the measurement of EF in preschool children [27, 28]. The task was divided into two stages. In the first stage, the tester showed the children two pictures of a similarity in one dimension (color, size, or content) and clearly illustrated the similarity to the child. Then, the third picture was presented, which had similarity in a different dimension with one of the first two pictures. The task required the children to choose which of the first two pictures had similarity with the third one. In the second stage, the testers showed three pictures to the children and asked them to choose two pairs of pictures with a similarity in a certain dimension, and the dimensions of the similarity in the two pairs should be different. The accuracy of the test was recorded to reflect children's cognitive flexibility.

#### 2.5.4. Physical Activity

Actigraph GT1M accelerometers were utilized to measure children's PA levels for a week. The accelerometers were worn on the right hip of the children with a waist belt. The accelerometers are valid instruments for the measurement of PA in preschool children [29]. The cut points recommended by Cauwenberghe et al. [30] were adopted to calculate children's time spent in moderate and vigorous PA (MVPA).

### 2.6. Statistical Analysis

The missing data were replaced using the multiple imputation techniques. Using IBM-SPSS 22.0 (IBM Inc., Armonk, NY), descriptive statistics were first calculated for all demographic and anthropometric measurements after which the pre- and posttest measures in SCA RT (ms), SCA accuracy (%), GNG RT, GNG accuracy, WMS accuracy, FIS accuracy, and MVPA for both intervention and control groups were presented as mean ± standard deviation. Internal consistency of each measure was calculated using Cronbach's alpha. Statistical analyses were conducted to examine the reaction time and response accuracy using 2 × 2 (group × test) repeated measures ANOVA. Partial eta-squared (*η*^2^) was applied to determine the effect size of EF test. An effect size of 0.02 was considered small; 0.13 medium; and 0.26 large [31]. The significance level was set at 0.05 for all statistical analyses.

## 3. Results

The characteristics of the participants are displayed in Table 1. A total of 26 girls (46.4%) and 31 boys (53.6%) were included in the current study. Notably, no significant differences were identified with age and body mass index between the control and intervention groups. The outcome measures of the intervention and control groups were presented in Table 2. Cronbach's alpha of the executive function task ranged from 0.71 to 0.85, which indicated that all measures had acceptable reliability. Specifically, intervention children's MVPA increased significantly from 44.9 min (5.3% of waking wear time) to 63.8 min (7.6%) after the intervention, whereas no significant difference was observed in the control group between pre- and posttest. Although the increase in MVPA in the intervention group was confirmed, no significant differences were seen in the SCA, GNG, WMS, and FIS tests between the intervention and control groups. This finding indicated that no significant improvements were found in the inhibitory control, working memory, and cognitive flexibility following a 10-week trampoline PA training in preschool children.

## 4. Discussion

Numerous cross-sectional studies have explored the association between PA and cognitive functions of children and adults [5, 32]. However, only a few intervention studies have been conducted using PA to promote preschool children's cognition. To the best of our knowledge, the current study was one of limited intervention studies investigating the effects of PA on the EF in Chinese preschool children. In the current study, compared with the young children in the control group, their counterparts in the intervention group received an extra 20 min of mini-trampoline PA in each school day for 10 weeks. The results indicated that children's MVPA was found to be improved after 10 weeks of trampoline training. Nevertheless, the study failed to find the positive influence of trampoline training on the EF in young children, including inhibitory control, working memory, and cognitive flexibility.

The length and amount of PA were regarded as the important moderators in the relationship between PA and cognitive functions [5]. Short length of intervention may not be sufficient to stimulate improvement in EF of preschool children. According to a recent systematic review, the length of most RCT studies on PA and cognitive functions of children ranges from 8 weeks to 9 months [5]. Compared with that, in previous RCT studies, the 10 weeks of intervention in the present study were relatively short. Nevertheless, positive effects of PA on cognitive functions are found in previous research followed by an 8-week and a 4-week interventions [14, 33, 34]. For example, Chang et al. [34] reported that a coordinative PA intervention, which is only 35-minute sessions two times per week for 8 weeks, can effectively increase the performance of EF in young children. In addition, a 4-week integrated PA (task-relevant PA included) has also been found to be beneficial to preschool children's cognitive functions [14, 33]. Therefore, relatively short intervention duration may not be the major reason for the insignificant EF improvement in the current study.

It was reported that high intensity PA maybe more beneficial for cognitive development in preschool children [35]. One plausible reason for the lack of significant improvement in cognitive function of preschool children in the current study might be that the intensity of PA intervention was not high enough to stimulate cognitive development. However, accelerometer data suggested that the MVPA time of intervention program in this study was similar to or longer than the MVPA time in previous PA interventions studies [14, 36]. This suggests that the intensity of the trampoline training was not the reason for the null results.

The mode of PA is another important factor that may affect the effectiveness of intervention. In the past decade, the effects of numerous types of PA on EF have been investigated. Some studies have shown that aerobic exercise and coordinative PA are beneficial to EF in preschool children [34, 35]. Nevertheless, a few studies have reported that no significant improvements in cognitive functions were observed after PA intervention [15]. In the current study, the benefit of trampoline, a favorite sport for children, to the EF of young children was investigated for the first time. Although the positive effects of trampoline on children's balance and motor skill have been confirmed [17], no significant improvements in inhibitory control, working memory, and cognitive flexibility of children were observed in this study. That is, the effective type of PA in improving the development of cognitive functions in children remains unclear. Recent studies have also investigated the effects of integrated PA combined with task-relevant PA. After merely 4 weeks of intervention, children in the integrated group exhibit better performance in working memories and learning outcomes than children in the nonintegrated PA and control groups [14, 33]. The results indicated that improved performance in cognitive functions can be achieved if the intervention is combined with PA and cognitive training. Nevertheless, the most appropriate type of PA for the development of cognitive function is still unclear. Additional studies are necessary to answer this important question.

EF is a complex psychological term that covers a series of cognitive abilities, including attentional control, cognitive flexibility, and goal setting [22]. The complicated composition of EF increases the difficulty in measurement, particularly among preschool children. Numerous psychological tasks have been developed to measure the EF of children, many of which have been applied to the field of PA and health. The benefits of PA on EF can be task specific, and variability in the measurements causes difficulty in synthesizing the results [5]. In particular, only small effects are found in most studies [37]. Therefore, the inconsistent results of RCT studies on PA intervention can be partly explained by the differences in the requirements and emphasis of the tests. In the current study, the SCA, GNG, WMS, and FIS tasks were employed to assess the inhibitory control, working memory, and cognitive flexibility of children. These tasks were particularly designed for 3 to 5-years-old children [20, 22, 38] and widely applied in the measurement of EF in young children in Western countries and China [39–41]. The results of the current study also indicated that the reliability of these EF tasks was acceptable. However, the instruments applied in the present study were modified based on classical executive function tests, which were different from those of the previous studies, and thus the inconsistent results obtained from this study could be partially due to the different EF tests applied.

Although no significant improvement in preschool children's EF was observed, the study found significant differences in PA as children in the intervention group showed increased MVPA more than the control group. PA is extremely important to the physical and mental development of young children. Taken together, the current study confirmed that PA did not exert deleterious effects on the cognitive functions of children [5].

Several limitations should be noted to inform future study. First, the sample size was relatively small and the intervention duration was relatively short. Therefore, the study may not be able to detect small effects. The strengths of this study may include the RCT research design and application of valid and reliable instruments for the measurement of EF in young children.

## 5. Conclusion

The data of this study suggests that a 10-week of trampoline PA intervention appears to be an effective PA tool in improving preschool children's PA, but this type of PA modality may not be sufficient to prompt the improvement of EF. Yet, the findings must be clarified in future larger and longer trials to discern the proper application of the trampoline PA for cognitive development of children. In detail, future research should continue to use RCT designs, to include large representative samples, longer interventions, increased doses, and, if possible, other cognitive measures.

## Figures and Tables

**Table 1:** Descriptive data for participants' demographic characteristics and physical activity manipulation.

	Control	Intervention	Total	*p* ^*∗*^
*n*	28	29	57	
age	4.47 ± 0.31	4.35 ± 0.28	4.40 ± 0.30	>0.05
Number of girls (%)	13 (46.4%)	13 (44.8%)	26 (45.6%)	>0.05
BMI	15.27 ± 0.93	15.18 ± 0.95	15.21 ± 0.94	>0.05

*Note*. BMI: body mass index;  ^*∗*^difference between control group and intervention group.

**Table 2:** Outcomes in EF and physical activity in intervention group and control group.

	Reliability	Baseline		After intervention		Repeated measure ANOVA		
		Intervention	Vontrol	Intervention	Control	*F*	*p*	*η* ^2^
Inhibitory control								
SCA RT (ms)	0.71	1741.6 ± 272.2	1817.6 ± 200.2	1779.0 ± 122.5	1775.3 ± 276.1	1.07	>0.05	0.04
SCA accuracy (%)	0.78	76.1 ± 16.2	75.4 ± 26.4	78.8 ± 14.3	78.8 ± 19.7	0.18	>0.05	0.01
GNG RT	0.72	1533.5 ± 256.9	1581.4 ± 211.7	1545.2 ± 213.8	1518.1 ± 286.9	0.62	>0.05	0.02
GNG accuracy	0.80	93.7 ± 7.4	92.8 ± 10.9	94.7 ± 4.8	93.7 ± 3.9	1.02	>0.05	0.03
Working memory								
WMS accuracy	0.85	75.3 ± 14.2	79.8 ± 12.8	73.3 ± 17.7	76.4 ± 18.3	0.05	>0.05	0.01
Cognitive flexibility								
FIS accuracy	0.73	76.8 ± 10.7	79.5 ± 14.0	80.6 ± 12.7	80.2 ± 10.5	0.19	>0.05	0.01
Physical activity								
MVPA (min)		44.8 ± 5.3	46.3 ± 7.2	63.8 ± 5.6	45.9 ± 5.1	18.98	<0.05	0.40

*Note*. SCA: spatial conflict arrows; GNG: animal Go/NoGo; WMS: working memory span; FIS: flexible item selection; RT: reaction time; MVPA: moderate and vigorous physical activity; *η*^2^: partial eta-squared.
